# 2,3-Bis(ethyl­sulfan­yl)-1,4,5,8-tetra­thia­fulvalene-6,7-dicarbonitrile

**DOI:** 10.1107/S1600536811028601

**Published:** 2011-07-23

**Authors:** Rui-bin Hou, Dong-feng Li

**Affiliations:** a School of Chemistry and Life Science, Changchun University of Technology, Changchun 130012, People’s Republic of China

## Abstract

In the title compound, C_12_H_10_N_2_S_6_, all non-H atoms, except for those in the ethyl groups, lie in the same non-crystallographic plane, with a r.m.s. deviation of 0.0366 (5) Å. In the crystal structure, mol­ecules are linked through weak C—H⋯N hydrogen bonds between methyl and cyano groups, forming centrosymmetric dimers. The dimers are arranged along the *a* axis, due to inter­molecular N⋯S [3.337 (4) Å] inter­actions.

## Related literature

For synthetic uses of dicyano-substituted tetra­thia­fulvalene derivatives, see: Chen *et al.* (2007 ▶); Leng *et al.* (2010 ▶). For a related structure, see: Jiang *et al.* (2010 ▶). For the synthesis of the title compound, see: Chen *et al.* (2005 ▶).
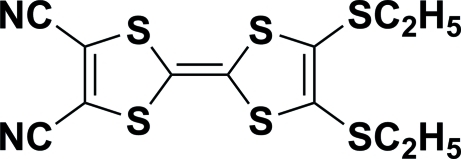


## Experimental

### 

#### Crystal data

C_12_H_10_N_2_S_6_
*M* *_r_* = 374.58
Triclinic, 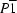

*a* = 7.8357 (16) Å
*b* = 8.9777 (18) Å
*c* = 12.618 (3) Å
α = 76.48 (3)°
β = 77.59 (3)°
γ = 73.20 (3)°
*V* = 815.8 (3) Å^3^
*Z* = 2
Mo *K*α radiation
μ = 0.83 mm^−1^
*T* = 293 K
0.15 × 0.13 × 0.12 mm

#### Data collection

Rigaku R-AXIS RAPID diffractometer
Absorption correction: multi-scan (*ABSCOR*; Higashi, 1995 ▶) *T*_min_ = 0.886, *T*_max_ = 0.907
8038 measured reflections
3689 independent reflections
3079 reflections with *I* > 2σ(*I*)
*R*_int_ = 0.022

#### Refinement

*R*[*F* ^2^ > 2σ(*F* ^2^)] = 0.031
*wR*(*F* ^2^) = 0.109
*S* = 1.15
3689 reflections
183 parameters
H-atom parameters constrained
Δρ_max_ = 0.37 e Å^−3^
Δρ_min_ = −0.42 e Å^−3^

### 

Data collection: *RAPID-AUTO* (Rigaku, 1998 ▶); cell refinement: *RAPID-AUTO*; data reduction: *CrystalStructure* (Rigaku/MSC, 2002 ▶); program(s) used to solve structure: *SHELXS97* (Sheldrick, 2008 ▶); program(s) used to refine structure: *SHELXL97* (Sheldrick, 2008 ▶); molecular graphics: *SHELXTL* (Sheldrick, 2008 ▶); software used to prepare material for publication: *SHELXL97*.

## Supplementary Material

Crystal structure: contains datablock(s) Global, I. DOI: 10.1107/S1600536811028601/bh2367sup1.cif

Structure factors: contains datablock(s) I. DOI: 10.1107/S1600536811028601/bh2367Isup2.hkl

Additional supplementary materials:  crystallographic information; 3D view; checkCIF report

## Figures and Tables

**Table 1:** Hydrogen-bond geometry (Å, °)

*D*—H⋯*A*	*D*—H	H⋯*A*	*D*⋯*A*	*D*—H⋯*A*
C10—H10*C*⋯N2^i^	0.96	2.73	3.659 (4)	164

Symmetry code: (i) 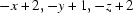.

**Fig. 1.**
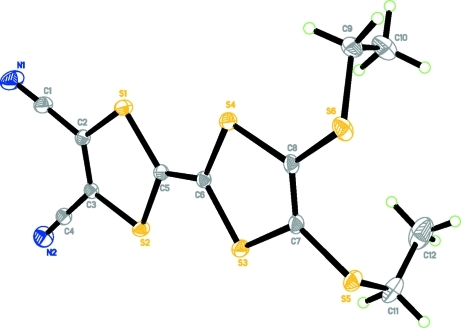
The crystal structure of the title compound, with displacement ellipsoids for non-H atoms drawn at the 20% probability level.

## supplementary crystallographic information

### Comment

Dicyano-substituted tetrathiafulvalene derivatives (TTFs) are key precursors for the preparation of the TTF-annulated prophyrazines. We have recently synthesized the symmetrical (Chen *et al.*, 2007) and the unsymmetrical TTF-annulated porphyrazines (Leng *et al.*, 2010) using such precursors. In this paper, we report the crystal structure of the title compound.

In the title compound (Fig. 1), all bond lengths and angles are in the normal ranges and comparable with those observed in a closely related compound (Jiang *et al.*, 2010). In the title compound, except for two ethyl groups, all atoms lie on the same plane. In the crystal, the molecules form dimers through weak intermolecular C—H···N hydrogen bonds (Table 1), and dimers are arranged along the *a* axis, due to N···S interactions.

### Experimental

The title compound was prepared according to the literature (Chen *et al.*, 2005). Single crystals suitable for X-ray diffraction were prepared by slow evaporation of a solution in a mixture of dichloromethane and petroleum ether, at room temperature.

### Refinement

C-bound H-atoms were placed in calculated positions (C—H 0.96 or 0.97 Å) and were included in the refinement in the riding model approximation, with *U*_iso_(H) = 1.5 *U*_eq_(C) for methyl groups and *U*_iso_(H) = 1.2 *U*_eq_(C) for methylene groups.

### Crystal data

**Table:**

C_12_H_10_N_2_S_6_	*Z* = 2
*M**_r_* = 374.58	*F*(000) = 384
Triclinic, *P*1	*D*_x_ = 1.525 Mg m^−^^3^
Hall symbol: -P 1	Mo *K*α radiation, λ = 0.71073 Å
*a* = 7.8357 (16) Å	Cell parameters from 3994 reflections
*b* = 8.9777 (18) Å	θ = 3.2–27.5°
*c* = 12.618 (3) Å	µ = 0.83 mm^−^^1^
α = 76.48 (3)°	*T* = 293 K
β = 77.59 (3)°	Block, black
γ = 73.20 (3)°	0.15 × 0.13 × 0.12 mm
*V* = 815.8 (3) Å^3^	

### Data collection

**Table:**

Rigaku R-AXIS RAPID diffractometer	3689 independent reflections
Radiation source: fine-focus sealed tube	3079 reflections with *I* > 2σ(*I*)
graphite	*R*_int_ = 0.022
ω scans	θ_max_ = 27.5°, θ_min_ = 3.2°
Absorption correction: multi-scan (*ABSCOR*; Higashi, 1995)	*h* = −10→10
*T*_min_ = 0.886, *T*_max_ = 0.907	*k* = −11→10
8038 measured reflections	*l* = −16→16

### Refinement

**Table:**

Refinement on *F*^2^	Primary atom site location: structure-invariant direct methods
Least-squares matrix: full	Secondary atom site location: difference Fourier map
*R*[*F*^2^ > 2σ(*F*^2^)] = 0.031	Hydrogen site location: inferred from neighbouring sites
*wR*(*F*^2^) = 0.109	H-atom parameters constrained
*S* = 1.15	*w* = 1/[σ^2^(*F*_o_^2^) + (0.0602*P*)^2^ + 0.1175*P*] where *P* = (*F*_o_^2^ + 2*F*_c_^2^)/3
3689 reflections	(Δ/σ)_max_ = 0.001
183 parameters	Δρ_max_ = 0.37 e Å^−^^3^
0 restraints	Δρ_min_ = −0.42 e Å^−^^3^
0 constraints	

### Fractional atomic coordinates and isotropic or equivalent isotropic displacement parameters (Å^2^)

**Table:**

	*x*	*y*	*z*	*U*_iso_*/*U*_eq_	
C1	1.3781 (3)	0.3004 (3)	1.17179 (18)	0.0471 (5)	
C2	1.2353 (2)	0.2956 (2)	1.11964 (14)	0.0346 (4)	
C3	1.0593 (2)	0.3581 (2)	1.15573 (14)	0.0325 (4)	
C4	0.9973 (3)	0.4385 (2)	1.24706 (16)	0.0404 (4)	
C5	1.0603 (2)	0.2358 (2)	0.99101 (14)	0.0312 (4)	
C6	1.0078 (2)	0.1834 (2)	0.91471 (14)	0.0314 (4)	
C7	0.8174 (3)	0.1324 (2)	0.78825 (14)	0.0362 (4)	
C8	0.9902 (3)	0.0668 (2)	0.74847 (14)	0.0348 (4)	
C9	1.2580 (3)	0.0150 (3)	0.56711 (17)	0.0514 (5)	
H9A	1.3429	−0.0101	0.6183	0.062*	
H9B	1.3115	−0.0489	0.5104	0.062*	
C10	1.2308 (4)	0.1859 (3)	0.5143 (2)	0.0673 (7)	
H10A	1.1425	0.2134	0.4662	0.101*	
H10B	1.3430	0.2042	0.4725	0.101*	
H10C	1.1895	0.2499	0.5705	0.101*	
C11	0.5366 (3)	0.3331 (3)	0.6882 (2)	0.0658 (7)	
H11A	0.4252	0.3457	0.6615	0.079*	
H11B	0.5078	0.3900	0.7489	0.079*	
C12	0.6628 (5)	0.4051 (4)	0.5971 (3)	0.1003 (13)	
H12A	0.7769	0.3859	0.6210	0.150*	
H12B	0.6122	0.5171	0.5789	0.150*	
H12C	0.6801	0.3584	0.5333	0.150*	
N1	1.4901 (3)	0.3032 (3)	1.21488 (19)	0.0733 (6)	
N2	0.9451 (3)	0.5027 (2)	1.31940 (17)	0.0632 (5)	
S1	1.28831 (6)	0.20402 (6)	1.00515 (4)	0.03914 (14)	
S2	0.89965 (6)	0.33925 (6)	1.08715 (4)	0.03802 (14)	
S3	0.77877 (6)	0.21620 (6)	0.90659 (4)	0.04141 (14)	
S4	1.15711 (6)	0.07338 (6)	0.81983 (4)	0.03804 (14)	
S5	0.62815 (7)	0.12625 (7)	0.73859 (4)	0.04650 (16)	
S6	1.05259 (8)	−0.03845 (7)	0.64039 (4)	0.04750 (16)	

### Atomic displacement parameters (Å^2^)

**Table:**

	*U*^11^	*U*^22^	*U*^33^	*U*^12^	*U*^13^	*U*^23^
C1	0.0377 (10)	0.0631 (13)	0.0461 (11)	−0.0110 (10)	−0.0063 (9)	−0.0232 (10)
C2	0.0360 (9)	0.0404 (9)	0.0320 (9)	−0.0122 (8)	−0.0083 (7)	−0.0094 (7)
C3	0.0369 (9)	0.0348 (9)	0.0290 (8)	−0.0107 (7)	−0.0058 (7)	−0.0090 (7)
C4	0.0420 (10)	0.0436 (10)	0.0369 (10)	−0.0073 (9)	−0.0071 (8)	−0.0136 (8)
C5	0.0324 (8)	0.0345 (9)	0.0288 (8)	−0.0090 (7)	−0.0058 (7)	−0.0081 (7)
C6	0.0341 (8)	0.0355 (9)	0.0274 (8)	−0.0107 (7)	−0.0068 (7)	−0.0070 (7)
C7	0.0404 (9)	0.0443 (10)	0.0301 (9)	−0.0177 (8)	−0.0111 (7)	−0.0050 (7)
C8	0.0434 (9)	0.0416 (9)	0.0255 (8)	−0.0183 (8)	−0.0084 (7)	−0.0060 (7)
C9	0.0471 (11)	0.0647 (14)	0.0430 (11)	−0.0095 (11)	0.0012 (9)	−0.0245 (10)
C10	0.0797 (18)	0.0730 (16)	0.0516 (14)	−0.0346 (14)	0.0086 (12)	−0.0144 (12)
C11	0.0585 (14)	0.0618 (15)	0.0846 (18)	−0.0060 (12)	−0.0410 (14)	−0.0121 (13)
C12	0.115 (3)	0.085 (2)	0.110 (3)	−0.050 (2)	−0.065 (2)	0.0395 (19)
N1	0.0473 (11)	0.1120 (18)	0.0767 (15)	−0.0167 (12)	−0.0174 (11)	−0.0453 (14)
N2	0.0704 (13)	0.0684 (13)	0.0527 (12)	−0.0068 (11)	−0.0075 (10)	−0.0303 (10)
S1	0.0314 (2)	0.0513 (3)	0.0399 (3)	−0.0088 (2)	−0.00407 (19)	−0.0216 (2)
S2	0.0303 (2)	0.0491 (3)	0.0386 (3)	−0.0089 (2)	−0.00466 (18)	−0.0176 (2)
S3	0.0336 (2)	0.0591 (3)	0.0365 (3)	−0.0109 (2)	−0.00710 (19)	−0.0180 (2)
S4	0.0348 (2)	0.0504 (3)	0.0336 (3)	−0.0103 (2)	−0.00648 (18)	−0.0162 (2)
S5	0.0454 (3)	0.0585 (3)	0.0465 (3)	−0.0236 (3)	−0.0173 (2)	−0.0084 (2)
S6	0.0633 (3)	0.0552 (3)	0.0346 (3)	−0.0267 (3)	−0.0028 (2)	−0.0185 (2)

### Geometric parameters (Å, °)

**Table:**

C1—N1	1.136 (3)	C8—S4	1.7606 (18)
C1—C2	1.430 (3)	C9—C10	1.496 (3)
C2—C3	1.352 (3)	C9—S6	1.810 (2)
C2—S1	1.7423 (19)	C9—H9A	0.9700
C3—C4	1.425 (2)	C9—H9B	0.9700
C3—S2	1.7314 (18)	C10—H10A	0.9600
C4—N2	1.132 (3)	C10—H10B	0.9600
C5—C6	1.346 (2)	C10—H10C	0.9600
C5—S2	1.7646 (19)	C11—C12	1.500 (4)
C5—S1	1.7673 (18)	C11—S5	1.800 (3)
C6—S4	1.7495 (19)	C11—H11A	0.9700
C6—S3	1.7543 (18)	C11—H11B	0.9700
C7—C8	1.348 (3)	C12—H12A	0.9600
C7—S5	1.7483 (18)	C12—H12B	0.9600
C7—S3	1.7569 (19)	C12—H12C	0.9600
C8—S6	1.7439 (19)		
			
N1—C1—C2	178.9 (3)	H9A—C9—H9B	107.7
C3—C2—C1	122.92 (17)	C9—C10—H10A	109.5
C3—C2—S1	117.99 (14)	C9—C10—H10B	109.5
C1—C2—S1	119.09 (15)	H10A—C10—H10B	109.5
C2—C3—C4	123.81 (17)	C9—C10—H10C	109.5
C2—C3—S2	118.14 (13)	H10A—C10—H10C	109.5
C4—C3—S2	118.05 (14)	H10B—C10—H10C	109.5
N2—C4—C3	178.8 (2)	C12—C11—S5	113.3 (2)
C6—C5—S2	120.78 (14)	C12—C11—H11A	108.9
C6—C5—S1	123.79 (15)	S5—C11—H11A	108.9
S2—C5—S1	115.42 (10)	C12—C11—H11B	108.9
C5—C6—S4	123.86 (14)	S5—C11—H11B	108.9
C5—C6—S3	121.49 (15)	H11A—C11—H11B	107.7
S4—C6—S3	114.62 (10)	C11—C12—H12A	109.5
C8—C7—S5	125.25 (15)	C11—C12—H12B	109.5
C8—C7—S3	117.19 (14)	H12A—C12—H12B	109.5
S5—C7—S3	117.34 (11)	C11—C12—H12C	109.5
C7—C8—S6	123.52 (14)	H12A—C12—H12C	109.5
C7—C8—S4	116.94 (14)	H12B—C12—H12C	109.5
S6—C8—S4	119.23 (11)	C2—S1—C5	94.04 (9)
C10—C9—S6	113.94 (17)	C3—S2—C5	94.40 (8)
C10—C9—H9A	108.8	C6—S3—C7	95.39 (9)
S6—C9—H9A	108.8	C6—S4—C8	95.48 (9)
C10—C9—H9B	108.8	C7—S5—C11	101.18 (10)
S6—C9—H9B	108.8	C8—S6—C9	102.91 (10)

### Hydrogen-bond geometry (Å, °)

**Table:**

*D*—H···*A*	*D*—H	H···*A*	*D*···*A*	*D*—H···*A*
C10—H10C···N2^i^	0.96	2.73	3.659 (4)	164.

Symmetry codes: (i) −*x*+2, −*y*+1, −*z*+2.
